# Nuclear factor (erythroid-derived 2)-like 2 counter-regulates thymosin beta-4 expression and primary cilium formation for HeLa cervical cancer cell survival

**DOI:** 10.1038/s41598-022-24596-6

**Published:** 2022-11-23

**Authors:** Jae-Wook Lee, Pham Xuan Thuy, Ja Hyun Koo, Eun-Yi Moon

**Affiliations:** 1 Department of Integrative Bioscience and Biotechnology, Sejong University, 209 Neungdong-Ro Kunja-Dong Kwangjin-Gu, Seoul, 05006 Republic of Korea; 2 College of Pharmacy and Research Institute of Pharmaceutical Sciences, Seoul National University, Seoul, 08826 Republic of Korea

**Keywords:** Cancer, Cell biology

## Abstract

We investigated the function of thymosin beta-4 (TB4) expression and primary cilium (PC) formation via the underlying Nrf2-dependent mechanism for cervical cancer cell (CC) survival under conditions of serum deprivation (SD). TB4 silencing was achieved using RNA interference. The percentage of PC formation was analyzed by immunofluorescence staining. Nrf2 expression was modified by the preparation of stable Nrf2-knockdown cells with shNrf2 and the overexpression of Nrf2 with pcDNA-Nrf2 plasmids. Gene expression was measured using reverse-transcription PCR, Gaussia luciferase assay, and western blotting. Cell viability was assessed using the MTT assay or CellTiter Glo assay. Reactive oxygen species (ROS) were detected with flow cytometry. CCs incubated in SD without fetal bovine serum remained viable, and SD increased PC formation and TB4 transcription. CC viability was further decreased by treatment with ciliobrevin A to inhibit PC formation or TB4-siRNA. SD increased ROS, including H_2_O_2_. N-acetylcysteine inhibited ROS production following H_2_O_2_ treatment or SD, which also decreased PC formation and TB4 transcription. Meanwhile, H_2_O_2_ increased PC formation, which was attenuated in response to TB4 siRNA. Treatment with H_2_O_2_ increased Nrf2 expression, antioxidant responsive element (ARE) activity, and PC formation, which were inhibited by the Nrf2 inhibitor clobestasol propionate. Nrf2 knockdown via expression of Tet-On shNrf2 enhanced ROS production, leading to increased PC formation and decreased TB4 expression; these effects were counteracted by Nrf2 overexpression. Our data demonstrate that Nrf2 counter-regulates TB4 expression and PC formation for CC survival under conditions of SD, suggesting cervical CC survival could be upregulated by PC formation via Nrf2 activation and TB4 expression.

## Introduction

Many cellular events are altered under conditions of serum deprivation (SD)^1^. SD inhibits the proliferation of HeLa cervical cancer cells (CCs)^2^. SD also induces apoptotic cell death through mitochondrial changes such as reactive oxygen species (ROS) production^3,4^. SD-induced apoptosis has been observed in many cell types, including CCs^5^. For instance, CC death by anticancer drugs was synergistically increased by SD to synchronize the cell cycle^5^. SD-triggered apoptosis is regulated by various signaling molecules^6–8^. However, how CC viability is regulated under conditions of SD is poorly understood.

Primary cilium prevalence is induced via the incubation of cultured cells with SD medium that regulates its ciliary stability in many cell types^9–12^. The PC is a microtubule-based non-motile signaling organelle that grows in a specific region of the plasma membrane and senses changes in nutrient levels^13^. When observed in quiescent and proliferating cells, the PC generally functions as a mechano-, osmo-, and chemosensory organelle that regulates cell cycle, differentiation, polarity, and migration during embryonic development, and maintains tissue and organ homeostasis^14,15^. The PC is assembled in the G0/G1 phase; its disassembly is initiated in the S phase and completed in the G2/M phase^16^. The molecules that regulate PC formation under conditions of SD, leading to CC survival, are poorly understood.

Thymosin beta-4 (TB4) may be a novel regulator of PC formation^17^ and it has an influence tumor growth^18^. TB4 is a naturally occurring 43-amino acid actin-sequestering protein. TB4 is ubiquitously expressed in most cell types except erythrocytes^19–22^. TB4 is associated with antioxidant enzyme expression^23,24^ and ROS production to control oxidative stress^25^. However, little is known regarding the control of TB4 expression and PC formation under conditions of SD, which result in CC viability.

ROS are increased under SD via incomplete mitochondrial oxidative phosphorylation^26^. Excessive ROS production contributes to various diseases, including cancer^27,28^. ROS are decomposed by antioxidant enzymes via activation of a cis-acting regulatory antioxidant responsive element (ARE) binding nuclear factor erythroid 2-like 2 (Nrf2)^29,30^. Nrf2 is a master transcription factor, which dissociates from Kelch-like ECH-associated protein 1 (Keap1) in the cytosol and translocates into the nucleus^30,31^. The Nrf2-ARE pathway is an attractive candidate for the SD-induced apoptotic response in various cell types^32,33^. The interplay between PC and Nrf2 may be important for human health and disease. It remains unknown whether NRF2 stimulates or negatively affects ciliogenesis^34^. It is also unclear whether SD-induced Nrf2 activation plays a direct or indirect role in PC formation and TB4 expression to regulate CC viability.

In this study, we investigated whether cell viability in SD conditions could be controlled by PC formation and TB4 expression using HeLa human cervical CCs. We demonstrated that PC formation is regulated by ROS-induced TB4 and Nrf2 under conditions of SD, ultimately regulating cell viability. These results suggest Nrf2 counter-regulates PC formation and TB4 expression.

## Methods

### Reagents

N-acetyl-L-cysteine(NAC), hydrogen peroxide(H_2_O_2_), MTT [3(4,5-dimethyl-thiazol-2-yl)-2,5-diphenyl tetrazolium bromide] and 4′,6-diamidno-2-phenylinole(DAPI) were purchased from the Sigma Chemical Co. (St. Louis, MO, USA). 2’,7’–dichlorofluorescin diacetate (DCF-DA) was purchased from Molecular Probe (Eugene, Oregon, USA). Rabbit antibodies which are reactive with Nrf2 (12,721) were from Cell Signaling Technology Inc. (Danvers, MA, USA). Mouse antibodies which are reactive with acetylated tubulin (T7451) or β-tubulin (T4026) were from Sigma-Aldrich Co. (St. Louis, MO, USA). Rabbit antibodies which are reactive with Arl13b (17,711–1-AP) were from Proteintech Group Inc. (Rosemont, IL, USA). Chicken anti-mouse IgG-Alexa 488 (A-21200) and goat anti-rabbit-Alexa 568 (A-11011) were obtained from Invitrogen (Calsbad, CA, USA). Except where indicated, all other materials are obtained from the Sigma Chemical Co. (St. Louis, MO, USA)^17^.

### Plasmids and siRNAs

Plasmids pcDNA3.1 was kindly provided from Prof. Young-Joo Jang, College of Dentistry, Dankook University (Cheon-An, Rep. of Korea), and pcDNA3.1-Nrf2 from Prof. Byung-Chul Kim, Division of Life Sciences, Kangwon National University (Chuncheon, Rep. of Korea).

Pre-designed promoter plasmids for TB4 (NM_021109) were obtained from GeneCopoeia Inc. (Rockville, MD, USA). TB4-promoter (HPRM20842) was 1242 bp (−2223 ~ −982) upstream from starting codon for TB4 transcription in Homo sapiens X BAC RP11-102M2 (AC139705.4)^17^. TB4 promoter was cloned at the sites between EcoRI and HindIII of Gaussia luciferase (Gluc) reporter plasmid vector, pEZX-PG02. ARE sequence, CAC CGT GAC TCA GCA ATT (3x) was cloned at the sites between BglII and HindIII of Gluc reporter plasmid vector, pEZX-PG02^17^.

Small interference(si) RNAs are customer-ordered to Bioneer (Daejeon, Rep. of Korea). Sequences of siRNAs for TB4 are as follows; sense: CCG AUA UGG CUG AGA A; anti-sense: UCG AUC UCA GCC AUA UCG G. AccuTarget™ negative control siRNA (SN-1001) was also purchased from Bioneer (Daejeon, Rep. of Korea)^17^.

### Cell culture^17^

HeLa human cervical cancer cells (ATCC # CCL-2) were obtained from Korea research institute of bioscience and biotechnology (KRIBB) cell bank (Daejeon, Rep.of Korea). Cells were cultured as monolayers in Dulbecco’s modified Eagle’s medium (DMEM) with supplement of 10% fetal bovine serum (FBS) (GIBCO, Grand Island, NY, USA), 2 mM L-glutamine, 100 units/ml penicillin and streptomycin (GIBCO, Grand Island, NY, USA). Cells were incubated at 37 °C in a humidified atmosphere of 5% CO_2_ maintenance. For the induction of primary cilium formation, cells were incubated in serum-deprived media with 0.1% FBS for 36 h^17^.

### Cytotoxicity assay^17^

Cell survival was quantified by using colorimetric assay with MTT to measure intracellular succinate dehydrogenase content^35^ or by using luminescence assay with CellTiter-Glo substrate to measure intracellular ATP content^18^. Confluent cells were cultured with various concentrations of each reagent for 24 h. Cells were then incubated with 50 μg/ml of MTT at 37 °C for 2 h. Formazan formed by MTT were dissolved in dimethylsulfoxide (DMSO). Optical density (OD) was measured at 540 nm^17^. For the CellTiter-Glo assay, cell cultures were treated with CellTiter-Glo substrate (Promega, Madison, WI). Luminescence was detected by using Lumet 3, LB9508 tube luminometer (Berthold Technologies GmbH & Co. KG, Bad Wildbad, Germany).

### Preparation of the stable Nrf2-knockdown cells^36^

The lentiviral vector of Nrf2-shRNA (shNrf2) was packaged into virus particles by the method reported previously^37^, which was provided by Sang-Min Jeon, Professor, College of Pharmacy, Ajou University (Gyeonggido, Rep. of Korea). 293T cells were transfected with a lentiviral vector using Lipofectamine® 2000 according to the recommended protocol on the Addgene website. Lentivirus-containing conditioned medium (LCCM) was aliquoted into 1 ml stock in each cryovial. Then, HeLa stable cells that do not express Nrf2 (tet-shNrf2, + Dox) were prepared as follows. Briefly, HeLa 1 × 10^5^ cells were incubated in each well of the 6-well plate overnight. Cell culture in 500 μl medium of each well was mixed with 1 ml LCCM and 1.2 μl polybrene (Millipore TR-1003-G). Culture medium was changed with 2 ml fresh medium containing 250 μg/ml hygromycin (Cayman 14,291). The infected shNrf2-positive control cells (tet-shNrf2, -DOX) were selected by the treatment with hygromycin every 3 days. Nrf2-knockdown (KD) cells were obtained and maintained by the treatment with 0.2 μg/ml doxycycline (Cayman 14,422) every 2 days.

### Detection of primary cilia^17^

Primary cilia in vitro were detected by immunostaining after cells were maintained in serum-deprived culture medium for 24–36 h^17^. Briefly, HeLa cells were grown on coverslip overnight and then incubated with serum-deprived DMEM with 0.1% FBS for 36 h. Cells were fixed with 4% paraformaldehyde for 10 min, washed three times with cold PBS and permeabilized with PBST (0.1% (v/v) Triton X-100 in PBS) for 10 min. Then, cells were washed with PBS three times, and incubated with rabbit Arl13b antibodies (1:1000) and/or monoclonal anti-acetylated tubulin antibodies diluted (1:1000) in PBST for 2 h at room temperature. After washing three times with PBS, cells were incubated with goat anti-rabbit IgG-Alexa 568 (1:2000) and/or chicken anti-mouse IgG-Alexa 488 diluted (1:1000) in PBST for 1 h at room temperature. Nucleus was visualized by staining cells with DAPI. After washing with PBS, coverslips with cells were mounted on slide glass. Primary cilia were observed and photographed at 1000X magnification under a fluorescence microscope (Nikon, Tokyo, Japan). PC frequency was evaluated by the blinded double scoring.

### Transfection of nucleic acids^17^

Each plasmid DNA, siRNAs for TB4 and AccuTarget™ negative contol siRNA were transfected into cells as follows^17^. Briefly, each nucleic acid and lipofectamine 2000 (Invitrogen, Calsbad, CA, USA) were diluted in serum-free medium and incubated for 5 min, respectively. The diluted nucleic acid and lipofectamine 2000 reagent were mixed by inverting and incubated for 20 min to form complexes. In the meanwhile, cells were stabilized by the incubation with culture medium without antibiotics and serum for at least 2 h prior to the transfection. Pre-formed complexes were added directly to the cells and cells were incubated for an additional 6 h. Then, culture medium was replaced with antibiotic and 10% FBS-containing DMEM and incubated for 24–48 h prior to each experiment.

### Gaussia luciferase assay for promoter activity^17^

HeLa cells were transfected with the ARE-Gluc or TB4-Gluc plasmids using lipofectamine 2000 (Invitrogen, Carlsbad, CA, USA) as described above. Then, cells were incubated for an appropriate time. Secreted Gluc reporter protein was obtained by the collection of culture-conditioned media after the indicated time intervals. Gluc activity of reporter protein was measured by Gaussia luciferase glow assay kit^®^ (Pierce Biotechnology, Rockford, IL, USA) including coelenterazine as a substrate for Gluc according to the manufacturer’s protocol. Luminescence was detected by using Lumet 3, LB9508 tube luminometer (Berthold Technologies GmbH & Co. KG, Bad Wildbad, Germany)^17^.

### H_2_O_2_ measurement^18^

The rate of H_2_O_2_ release was measured by the changes in fluorescence of scopoletin as reported previously^38,39^. Fluorescent scopoletin is changed into non-fluorescent materials by H_2_O_2_ production during the incubation. Briefly, cells were incubated with various concentrations of FBS for 12 or 24 h. The cultures were washed 3 times with PBS. The assay mixture (prewarmed to 37 °C) was prepared immediately before use from stock solutions and consisted of 30 μM scopoletin and 1 mM NaN_3_ in Krebs-Ringer phosphate buffer (KRP) supplemented with 5.5 mM glucose. KRP was constituted with 129 mM NaCl, 4.86 mM KCl, 0.54 mM CaCl_2_, 1.22 mM MgSO_4_, 15.8 mM sodium phosphate, pH 7.35, 300–315 mosM. Scopoletin was prepared as a 1 mM solution in KRP by dissolution for 24 h at 37 °C, sterile filtered and stored at 4 °C in the dark. Immediately after the addition of the assay mixture into the wells (100 μl/well), the fluorescence was measured on microplate fluorometer (SpecraFluor plus, TECAN, Alexandria, Austria) with the excitation at 360 nm and the emission at 460 nm. Then, the plate was transferred to the 37 °C incubation chamber and maintained for 60 min. The fluorescence (F) in each well was measured again and the fold changes for the results were calculated as below.

$${\text{Fold }}\;{\text{changes }} = \frac{{{\text{Fluorescence}}\;{\text{ difference}}\; \, \left( {{\text{F}}_{{{6}0\;{\text{ min}}}} - {\text{ F}}_{{0 \, \;{\text{min}}}} } \right)\;{\text{ in }}\;{\text{sample }}\;{\text{group}}}}{{{\text{Fluorescence}}\;{\text{ difference }}\;\left( {{\text{F}}_{{{6}0\;{\text{ min}}}} - {\text{ F}}_{{0 \, \;{\text{min}}}} } \right)\;{\text{ in }}\;{\text{control}}\;{\text{ group}}}}$$

### ROS measurement^35^

To measure the level of intracellular reactive oxygen species (ROS), cells were incubated with or without 10 μM DCF-DA at 37 °C for 30 min. Fluorescence intensity of 10,000 cells was analysed by FACSCalibur™ (Becton Dickinson, San Joes, CA, USA)^35^.

### Reverse transcription polymerase chain reaction (RT-PCR)^17^

Total RNA was extracted by using TRizol reagent (Invitrogen, Calsbad, CA, USA). Complementary DNA (cDNA) was synthesized from 1 μg of isolated total RNA, oligo-dT_18_, and superscript reverse transcriptase (Bioneer, Daejeon, Rep. of Korea) in a final volume of 20 μl. For standard PCR, 1 μl of template cDNA was amplified with Taq DNA polymerase. PCR amplification was performed with 30 ~ 35 thermocycles for 30 s at 95 °C, 30 s at 55 °C, and 60 s at 72 °C using oligonucleotide primers specific for human TB4 (sense: ACA AAC CCG ATA TGG CTG AG; anti-sense: CCT CCA AGG AAG AGA CTG AA), GAPDH (sense: GAA GGT GAA GGT CGG AGT C; anti-sense: GAA GAT GGT GAT GGG ATT TC) and actin (sense: GTC ACC AAC TGG GAC GAC AT; anti-sense: GCA CAG CCT GGA TAG CAA CG). Amplified PCR products were separated by 1.0–2.0% agarose gel electrophoresis and detected on Ugenius 3^®^ gel documentation system (Syngene, Cambridge, United Kingdom)^17^.

### Western blotting^17^

Cells were lysed in ice-cold RIPA buffer (Triton X-100,) containing protease inhibitor (2 μg/ml aprotinin, 1μMpepstatin, 1 μg/ml leupeptin, 1 mM phenylmetylsufonyl fluoride (PMSF), 5 mM sodium fluoride (NaF) and 1 mM sodium orthovanadate (Na_3_VO_4_)). The protein concentration of the sample was measured using SMART™ BCA protein assay kit (Pierce 23,228) from iNtRON Biotech. Inc. (Seoul, Rep. of Korea). The same amount of heat-denatured protein in sodium dodecyl sulfate (SDS) sample buffer was separated in sodium dodecyl sulfate polyacrylamide gel electrophoresis (SDS-PAGE), and then transferred to nitrocellulose membrane by using electro blotter. Equal amount of loaded sample on the membrane was verified by Ponceau S staining. In some cases, membranes were cut in accordance with each target protein size prior to hybridization with antibodies. The membrane was incubated with blocking solution [5% non-fat skim milk in Tris-buffered saline with Tween 20 (TBST)], and then followed by incubation with the specific primary antibodies. Horse radish peroxidase (HRP)-conjugated or IRdye-conjugated secondary antibody was used for target-specific primary antibody. Immuno-reactive target bands were visualized by the reaction with enhanced chemiluminescence (ECL-PS250) (Dongin LS, Seoul, Rep. of Korea) on X-ray film (Agfa HealthCare, Seol, Rep. of Korea) or by the detection of IRdye with Odyssey CLx Infrared Imaging System (LI-COR Biosciences, Lincoln, NE, Germany), respectively^17^. Original images of full-length blots were included in the Supplementary Information file.

### Statistical analysis

Experimental differences were verified for statistical significance using ANOVA and Student’s t-test. *P* value of < 0.05 and < 0.01 was considered to be significant as compared to control group for each experiment.

## Results

### Serum deprivation influences cell viability, primary cilium formation, and TB4 expression

**Figure 1:**
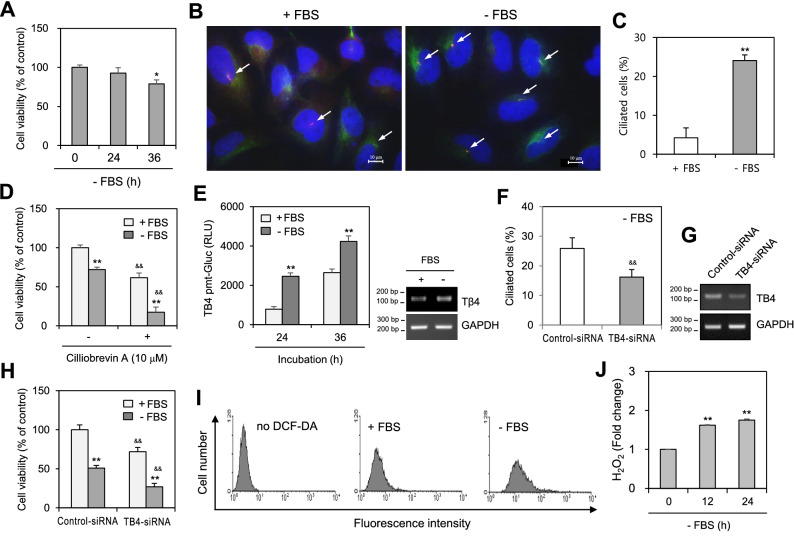
Serum-deprivation without fetal bovine serum (-FBS) affected cell viability, primary cilia formation, the expression of thymosin beta-4 and reactive oxygen species production. (**A**–**C**) HeLa cells were incubated in serum-deprived media without FBS. Cell viability was measured by MTT assay (**A**). The cells were fixed and stained with antibody against Ac-tubulin (green) and Arl13b (red). Nucleus was stained with DAPI (blue). The representative image of primary cilia was observed with 1000X magnification under fluorescence microscope. White arrows indicated primary cilia (**B**). The ciliated HeLa cells out of more than 1,000 cells in the presence (white) or absence (grey) of FBS were counted. PC frequency was evaluated by the blinded double scoring (**C**). (**D**) The cells were incubated in the presence or absence of ciliobrevin A under serum-deprived condition for 36 h. Cell viability was measured by MTT assay. (**E**) HeLa cells were transfected with pEZX-PG02-TB4-promoter Gaussia luciferase (Gluc) plasmid and incubated for up to 36 h. The activity of Gluc in cultured media was measured with luminometer using Gluc substrate (E, left). Expression level of TB4 was measured by RT-PCR (E, right). (**F**–**H**) Cells were transfected with AccuTarget negative control-siRNA or TB4-siRNA for 24 h. The cells were fixed and stained with antibody against Ac-tubulin (green) and Arl13b (red). Nucleus was stained with DAPI (blue). The ciliated cells out of more than 1000 cells in the presence (white) or absence (grey) of FBS were counted. PC frequency was evaluated by the blinded double scoring (**F**). The mRNA expression of TB4 was detected by RT-PCR (**G**). Cell viability was measured by CellTiter Glo assay (**H**). (**I**, **J**) Cells were incubated under serum-deprived condition for up to 24 h. Cells were treated with DCF-DA and ROS production was measured by FACS analysis (**I**). H_2_O_2_ production was measured with the changes in fluorescence of scopoletin (**J**). Each result was the representative of experiments performed at least four times and each experiment was performed with four samples (n = 4) in the same group. Data in bar graphs represents the means ± SD. *p < 0.05, **p < 0.01; significantly different from 5% FBS-treated (**A**, **C**–**E**, **H**) or 0% FBS-untreated (**J**) control group. ^&&^*p* < 0.01; significantly different from ciliobrevin A-untreated group (**D**) or control-siRNA-treated (**F**, **H**) group with either 5% or 0% FBS.

SD induces apoptosis of many cell types^5^, while TB4 induces PC formation and tumor growth^17,18^. We also examined the effects of SD on cell viability, PC formation, and TB4 expression. When cells were incubated in SD media without fetal bovine serum (FBS) for 36 h, cell viability, as measured using the MTT assay, was decreased (Fig. 1A). Cells were stained to cilium marker proteins, Arl13b and/or Ac-tubulin, to find PC. Marker proteins were detected by the observation of each cell under the fluorescence microscope. Arl13b and Ac-tubulin were co-localized in PC (Supplementary Fig. S1). An increase in PC frequency was detected in HeLa cells incubated under SD conditions (Fig. 1B,C). This was confirmed by the treatment of ciliogenesis inhibitor, ciliobrevin A, which resulted in a decrease of approximately 70% cell viability by the application of SD condition as compared to the inhibition of about 28% in ciliobrevin A-untreated control (Fig. 1D). Increased TB4 transcription was measured based on TB4 promoter activity (Fig. 1E, left) and its transcription (Fig. 1E, right) in response to incubation in SD medium. Knockdown (KD) of TB4 expression via TB4 siRNA resulted in a 40% decrease in PC frequency (Fig. 1F,G), leading to inhibition of cell viability by approximately 60% in response to incubation in SD medium compared to that in TB4-KD group with FBS (Fig. 1H). ROS production was detected using fluorescence-activated cell sorting analysis (Fig. 1I) and H_2_O_2_ levels were observed via changes in the fluorescence of scopoletin (Fig. 1J). The results suggest that SD-induced cell death may be related to TB4-associated PC formation.

### ROS are associated with PC formation and TB4 expression

**Figure 2:**
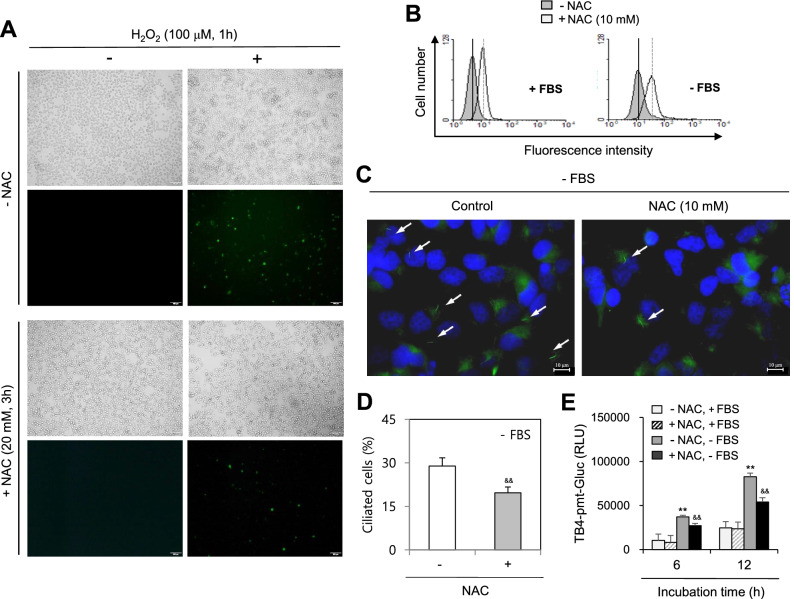
N-acetylcysteine (NAC) decreased reactive oxygen species production, primary cilia formation and the expression of thymosin beta-4 under serum-deprived (SD) condition. (**A**) Cells were treated with 100 μM H_2_O_2_ in the absence or presence of NAC. Cells were incubated with DCF-DA and ROS production was observed under the fluorescence microscope. (**B**–**D**) Cells were incubated under SD condition in the absence or presence of NAC. Cells were treated with DCF-DA and ROS production was measured by FACS analysis. Vertical dotted lines and solid lines indicate mean fluorescence intensity (MFI) for the increase and the inhibition of ROS production, respectively (**B**). Cells were fixed and stained with antibody against Ac-tubulin (green) and DAPI (blue). The representative image of primary cilia was observed with 400X magnification under fluorescence microscope. White arrows indicated primary cilia (**C**). The ciliated HeLa cells out of more than 1000 cells in the absence (white) or presence (grey) of NAC were counted. PC frequency was evaluated by the blinded double scoring (**D**). (**E**) HeLa cells were transfected with pEZX-PG02-TB4-promoter Gaussia luciferase (Gluc) plasmid and incubated with 5% or 0% FBS for up to 12 h. The activity of Gluc in cultured media was measured with luminometer using Gluc substrate. Each result was the representative of experiments performed at least four times and each experiment was performed with four samples (n = 4) in the same group. Data in bar graphs represents the means ± SD. **p < 0.01; significantly different from 5%-treated control group (**E**). ^&&^*p* < 0.01; significantly different from NAC-untreated group in 0% FBS (**D**, **E**).

**Figure 3:**
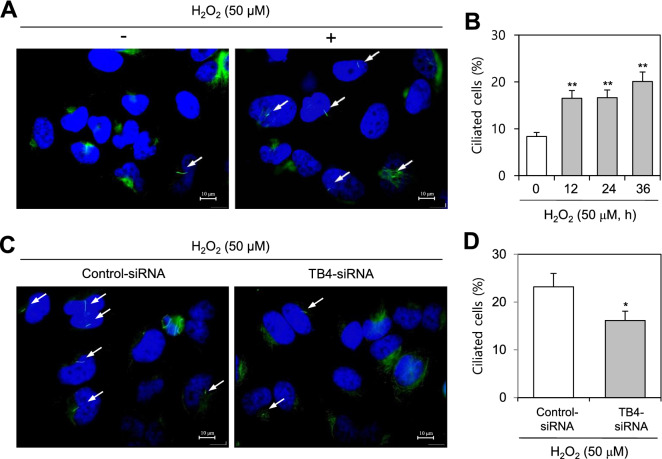
H_2_O_2_ increased primary cilium formation in HeLa cells. (**A**–**D**) HeLa cells were incubated with 50 μM H_2_O_2_ for up to 36 h (**A**, **B**). Cells were transfected with AccuTarget negative control-siRNA or TB4-siRNA for 24 h. Then, cells were incubated with 50 μM H_2_O_2_ (**C**, **D**). The cells were fixed and stained with antibody against Ac-tubulin. The representative image of primary cilia was observed with 400X magnification under fluorescence microscope. White arrows indicated primary cilia (**A**, **C**). The ciliated cells out of more than 1,000 cells in the absence (white) or presence (grey) of H_2_O_2_ were counted. PC frequency was evaluated by the blinded double scoring (**B**, **D**). Each result was the representative of experiments performed at least four times and each experiment was performed with four samples (n = 4) in the same group. Data in bar graphs represents the means ± SD. *p < 0.05, **p < 0.01; significantly different from H_2_O_2_-untreated control cells (**B**) or control-siRNA-treated and H_2_O_2_-treated control cells (**D**).

We assessed whether SD-induced ROS could affect PC formation and TB4 expression. Given that N-acetylcysteine (NAC) treatment inhibited the number of ROS-positive cells following treatment with H_2_O_2_ (Fig. 2A), the decrease in SD-induced ROS production was confirmed by treatment with NAC. Mean fluorescence intensity (MFI) for SD-induced increase of ROS was indicated by dotted lines, which was inhibited by NAC-treatment indicated in solid lines (Fig. 2B). NAC treatment also decreased PC formation in HeLa cells incubated under SD conditions by approximately 40% (Fig. 2C,D). Moreover, TB4 promoter activity under SD was reduced by NAC treatment (Fig. 2E), suggesting PC formation might be controlled by the SD-induced increase in ROS and TB4 promoter activity. We re-examined our data following treatment with H_2_O_2_. ROS-induced PC formation was confirmed by a 1.0–1.3-fold increase in PC frequency in the H_2_O_2_-treated group (Fig. 3A,B). When TB4 siRNA-treated cells were incubated with 50 μM H_2_O_2_ for 24 h, ROS-induced PC formation by TB4 expression was demonstrated by a 35% decrease in PC frequency in the TB4 siRNA-treated and H_2_O_2_-treated groups (Fig. 3C,D). These results suggest SD-induced PC formation could be regulated by ROS production and TB4 expression, which might be associated with cell survival in response to incubation in SD medium.

### ROS-increased Nrf2-ARE activity is related to PC formation and TB4 transcription

**Figure 4:**
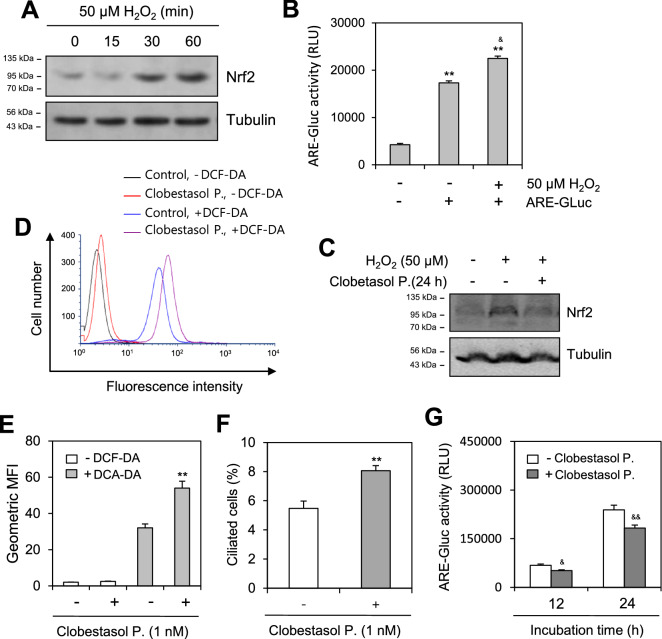
Effect of H_2_O_2_ and Nrf2 on primary cilia formation. (**A**) Cells were treated with 50 μM H_2_O_2_ for 15, 30 and 60 min. Cell lysates were prepared from each sample and Nrf2 proteins were detected by western blotting. (**B**) HeLa cells were transfected with antioxidant response element (ARE)-Gaussia luciferase (Gluc) plasmid DNA and incubated in the absence or presence of 50 μM H_2_O_2_. ARE-Gluc activity in cultured media was measured with luminometer using Gluc substrate. (**C**) Cells were treated with 50 μM H_2_O_2_ in the absence or presence of clobestasol propionate (P.). Cell lysates were prepared from each sample and Nrf2 proteins were detected by western blotting. (**D**–**F**) Cells were incubated in the absence or presence of clobestasol P under SD condition. Cells were treated with DCF-DA and ROS production was measured by FACS analysis (**D**). Geometric mean fluorescence intensity (MFI) in the absence (white) or presence (grey) of clobestasol P. was analyzed by WinMDI 2.8 for each sample (**E**). The cells were fixed and stained with antibody against Ac-tubulin. The number of ciliated HeLa cells out of more than 1000 cells in the absence (white) or presence (grey) of clobestasol P. were counted. PC frequency was evaluated by the blinded double scoring (**F**). (**G**) HeLa cells were transfected with pEZX-PG02-TB4-promoter Gaussia luciferase (Gluc) plasmid and incubated with SD condition in the absence (white) or presence (grey) of clobestasol P. for up to 24 h. The activity of Gluc in cultured media was measured with luminometer using Gluc substrate. Each result was the representative of experiments performed at least four times and each experiment was performed with four samples (n = 4) in the same group. Processing (such as changing brightness and contrast) is applied equally to controls across the entire image. Original images of full-length blots were included in the Supplementary Information file (**A**, **C**). Data in bar graphs represents the means ± SD (**B**, **E**–**G**). **p < 0.01; significantly different from H_2_O_2_-untreated and ARE-untransfected group (**B**) or SD-treated and clobestasol P.-untreated group (**E**, **F**). ^&^*p* < 0.05, ^&&^*p* < 0.01; significantly different from H_2_O_2_-untreated and ARE-transfected group (**B**) or clobestasol P.-untreated group (**G**).

As Nrf2 is a master transcription factor that activates ARE to induce the expression of antioxidant enzymes^29–31^, we tested whether ROS-increased Nrf2-ARE activity is related to PC formation and TB4 transcription. As H_2_O_2_ increased Nrf2 protein (Fig. 4A) and ARE activity (Fig. 4B), we examined the role of Nrf2 on PC formation and TB4 transcription using clobestasol propionate (P.) to inhibit Nrf2 protein levels (Fig. 4C). Intracellular ROS, detected by flow cytometry (Fig. 4D), were increased by approximately 45% following treatment with clobestasol P., which was estimated from the geometric mean fluorescence intensity (MFI) (Fig. 4E). Clobestasol P. treatment increased PC formation by approximately 35% (Fig. 4F), confirming that Nrf2 expression could reduce the prevalence in PC formation by H_2_O_2_ treatment (Fig. 3B). However, Clobestasol P. treatment reduced TB4 transcriptional activity by 25% (Fig. 4G). Our data demonstrate that Nrf2-ARE regulates SD-induced PC formation and TB4 expression.

### Nrf2 counter-regulates PC formation and TB4 expression

**Figure 5:**
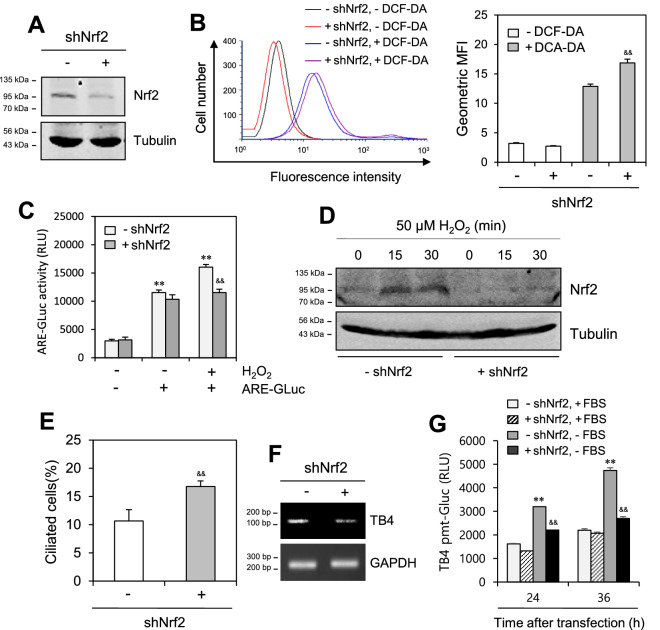
Thymosin beta-4 expression was inhibited in Nrf2-knockdown (KD) cells. (**A**–**G**) HeLa cells were treated with retroviral shNrf2. Then, shNrf2-positive stable cells were selected by the treatment with hygromycin. Nrf2-KD was induced by the incubation with doxycycline(Dox) for 2 days. Nrf2 proteins were detected by western blotting (**A**). Wildtype (WT) control and Nrf2-KD HeLa cells were treated with DCF-DA. ROS production was measured by FACS analysis (**B**, left). Geometric mean fluorescence intensity (MFI) was analyzed by WinMDI 2.8 for each sample (**B**, right). WT control and Nrf2-KD HeLa cells were transfected with antioxidant response element (ARE)-Gaussia luciferase (Gluc) plasmid DNA and incubated in the absence or presence of 50 μM H_2_O_2_. ARE-Gluc activity in cultured media was measured with luminometer using Gluc substrate (**C**). WT control and Nrf2-KD HeLa cells were incubated with 50 μM H_2_O_2_ for 15 and 30 min. Nrf2 proteins were detected by western blotting (**D**). The cells were fixed and stained with antibody against Ac-tubulin. The ciliated cells out of more than 1000 cells were counted in control (white) and Nrf2-knockdown (grey) group. PC frequency was evaluated by the blinded double scoring (**E**). TB4 transcripts were detected with RT-PCR (**F**). WT control and Nrf2-KD HeLa cells were transfected with pEZX-PG02-TB4-promoter Gaussia luciferase (Gluc) plasmid and incubated in the presence or absence of FBS. The activity of Gluc in cultured media was measured with luminometer using Gluc substrate (**G**). Each result was the representative of experiments performed at least four times and each experiment was performed with four samples (n = 4) in the same group. Processing (such as changing brightness and contrast) is applied equally to controls across the entire image. Original images of full-length blots were included in the Supplementary Information file (**A**, **D**). Data in bar graphs represents the means ± SD. **p < 0.01; significantly different from H_2_O_2_-untreated and ARE-untransfected or -transfected group (**C**) or 5% FBS-treated group (**G**). ^&^p < 0.05; ^&&^p < 0.01, significantly different from shNrf2-untreated WT control cells (**B**, right, **C**, **E**, **G**).

**Figure 6:**
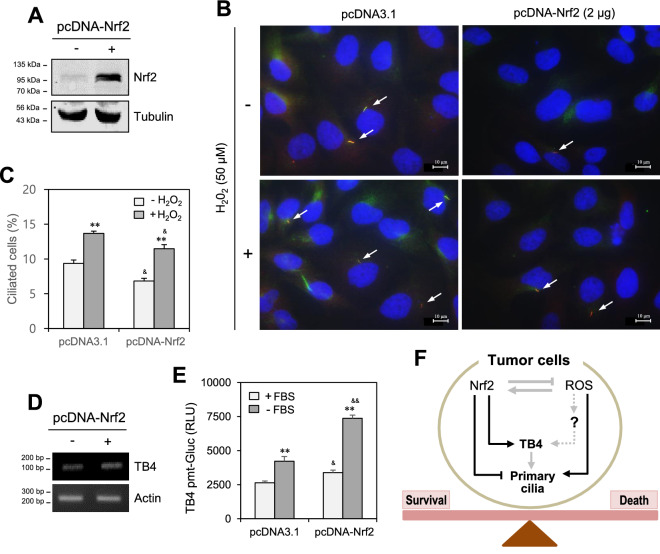
Thymosin beta-4 expression was enhanced by Nrf2-overexpressed cells. (**A**–**D**) Cells were transfected with pcDNA3.1 or pcDNA-Nrf2 plasmid DNA for 24 h. Nrf2 proteins were detected by western blotting. Processing (such as changing brightness and contrast) is applied equally to controls across the entire image. Original images of full-length blots were included in the Supplementary Information file (**A**). HeLa cells were treated with 50 μM of H_2_O_2_ for 24. Cells were fixed and stained with antibody against Ac-tubulin (green), Arl13b (red) and DAPI (blue). The representative image of primary cilia was observed with 1000X magnification under fluorescence microscope. White arrows indicated primary cilia (**B**). The number of ciliated HeLa cells out of more than 1000 cells were counted. PC frequency was evaluated by the blinded double scoring (**C**). The mRNA expression of TB4 was detected by RT-PCR (**D**). (**E**) pEZX-PG02-TB4-promoter Gaussia luciferase (Gluc) plasmids were co-transfected with pcDNA3.1 or pcDNA-Nrf2 plasmid DNA into HeLa cells. Then, cells were incubated in the presence or absence of FBS. The activity of Gluc in cultured media was measured with luminometer using Gluc substrate. Each result was the representative of experiments performed at least four times and each experiment was performed with four samples (n = 4) in the same group. Data in bar graphs represents the means ± SD. *p < 0.05, **p < 0.01; significantly different from H_2_O_2_-untreated (**C**) or 5% FBS-treated group (**E**). ^&^*p *< 0.05; ^&&^*p* < 0.01; significantly different from pcDNA3.1-transfected and H_2_O_2_-untreated or -treated group (**C**) or 5% FBS- or 0% FBS-treated group (**E**). (**F**) Schematic summary for primary cilia formation by Nrf2 and ROS through TB4 expression to regulate CC survival and death. While Nrf2 conversely regulated primary cilium formation and TB4 expression in HeLa human cervical CCs (solid line). ROS also up-regulate TB4 expression through unknown another factors (marked as question mark and grey dotted line). Our findings were indicated by black solid lines. Pathway already known was indicated by grey solid lines.

We examined how Nrf2 regulates PC formation and TB4 expression by KD or overexpression of Nrf2. When HeLa cells were treated with retroviral shNrf2, Nrf2-KD was detected via 2-day incubation with doxycycline following selection with hygromycin (Fig. 5A). A small increase in intracellular ROS was detected using flow cytometry (Fig. 5B, left). The MFI in Nrf2-KD cells was approximately 25% higher than that in wild-type control cells (Fig. 5B, right). Nrf2-KD was confirmed by a 25% reduction in ARE activity in response to H_2_O_2_ treatment (Fig. 5C) and H_2_O_2_-induced Nrf2 protein (Fig. 5D). Control and Nrf2-KD HeLa cells were incubated with 50 μM H_2_O_2_ for 15 and 30 min. Nrf2 proteins were detected using western blotting (Fig. 5D). While Nrf2-KD increased PC formation by approximately 45% (Fig. 5E), it reduced TB4 expression (Fig. 5F) and transcriptional activity 40–49% under SD conditions (Fig. 5G). The effect of Nrf2 was confirmed by Nrf2 overexpression (Fig. 6A), confirming that Nrf2 expression could reduce the prevalence in PC formation by H_2_O_2_ treatment (Fig. 3B). While Nrf2 overexpression decreased PC formation 30–40% (Fig. 6B,C), it enhanced TB4 expression (Fig. 6D) and transcriptional activity approximately 1.4–2.2-fold under SD conditions (Fig. 6E). Our data demonstrate that Nrf2 counter-regulates SD-induced PC formation and TB4 expression, suggesting Nrf2 can control CC viability via PC formation and TB4 expression (Fig. 6F).

## Discussion

SD changes various cellular conditions^1^. Although SD induces proliferation inhibition^2^ and apoptotic cell death^3,4^, CCs continue to grow under SD conditions. SD-mediated cellular events are regulated by various signaling molecules^6–8^. It remains unclear which signaling molecules are associated with SD-triggered events, controlling CC survival. Microtubule-based non-motile PC regulates cell cycle, differentiation, polarity, and migration and maintains tissue and organ homeostasis^13–15^. TB4, a naturally occurring actin-sequestering protein^19–22^, could be a novel regulator of PC formation^17^ to enhance tumor growth^18^. TB4 also controls antioxidant enzyme expression and oxidative stress^23–25^. SD-induced ROS are inactivated by antioxidant enzymes; expression of these enzymes is increased by ARE binding of Nrf2^29,30^. Here, we investigated how Nrf2 could control the nexus between PC formation and TB4 expression, regulating CC viability. Our data showed that PC formation was downregulated by H_2_O_2_-Nrf2 and upregulated by H_2_O_2_-TB4 under SD conditions, affecting cell viability. This suggests Nrf2 counter-regulates PC formation and TB4 expression for cervical CC survival.

It is unclear how CC survival and death are controlled under conditions of SD. PC formation, a representative SD-induced event in many types of cultured cells^9–11,40^, has been shown to inhibit CC proliferation^41^ and has been correlated with some proliferative CCs^42^. TB4 and ROS are representative SD-induced molecules for PC formation and tumor growth^3,17,18^. Our data show that CC viability under SD conditions was reduced by inhibition of PC formation (Fig. 1D). TB4 expression regulated PC formation, which was dependent on ROS production (Figs. 1 and 2), including H_2_O_2_ (Fig. 3). It will be important to define the molecules involved in SD and how they affect PC formation and TB4 expression.

Oxidative stress caused by excessive ROS production contributes to various diseases^27,28^. Nrf2 is a master transcription factor involved in antioxidant and detoxification responses^43,44^. Oxidant injury can be prevented by Nrf2 induction^45^. We showed that H_2_O_2_ induced Nrf2, whereas inhibition of Nrf2 with clobestasol P. increased PC formation (Fig. 4). While Nrf2-KD increased ROS production and PC formation, it decreased TB4 expression in cells expressing shNrf2 (Fig. 5). By contrast, Nrf2 overexpression reduced PC formation but enhanced TB4 expression (Fig. 6). Our data demonstrate that TB4 may link ROS production and PC formation. These results suggest Nrf2 counter-regulates PC formation and TB4 expression in response to ROS.

SD alters various cellular events and signaling molecules. In the face of apoptosis, SD increased activity of the PI3K/Akt and MEK/ERK1/2 pathways^8^ and inhibited activity of the p38/Bcl-2 pathway^6^. SD decreased phosphorylation of ERK1/2 but increased phosphorylation of JNK1/2 and p38^46^. Moreover, SD significantly increased expression of the autophagy-related proteins Atg5, Beclin1, LC3, and p62/SQSMT1^47^. In addition, SD triggered Ca^++^ mobilization from the endoplasmic reticulum and caspase-12 activation^7^. Therefore, it will be important to further define the relationship between Nrf2 and other signaling molecules involved in PC formation and TB4 expression.

Many molecules on the PC membrane regulate cell proliferation, migration, and differentiation^48^. Membrane transporters and various receptors can transduce extracellular signals into cells^13,48,49^. PC is associated with diverse signaling pathways mediated by hedgehog, wigless, hippo (Salvador-Warts-Hippo), JAK/STAT, TRPV4, cAMP/cGMP, and mTOR^14,15^. Thus, it is possible for ROS (e.g., H_2_O_2_) to stimulate those signaling pathways for TB4 expression and PC formation. Further studies are needed to define how those proteins regulate CC viability under SD conditions.

TB4 is an actin-sequestering protein that interacts with monomeric globular actin^22^ to regulate actin cytoskeleton dynamics. Thus, TB4 may be implicated in CC survival via PC formation. TB4 induces anti-apoptosis and paclitaxel resistance via inhibition of caspase-3 activation^50^, ROS production, and hypoxia-inducible factor-1α stabilization^25,51,52^. TB4 also regulates glycogen synthase kinase-3, Rac1-GTPase, and Rap1-GTPase^12,53,54^. Further studies are need to define the relationship between TB4-associated molecules, ROS-Nrf2, and PC formation. It is also required to explore roles of other signalling molecules to strengthen causative effects and to link PC formation to CC viability.

In addition, recent studies indicate that actin polymerization by direct and indirect involvement of actin regulators impacts ciliogenesis. Defective actin dynamics cause defects in ciliogenesis^55^. Primary cilium elongation was induced by the enrichment of many actin-binding proteins inside cilium and actin depolymerization^56^. While actin stress fibers inhibit cilia formation, their disruption by cytochalasin D elongates cilia length and number^57–59^. The interaction between cilia and actin regulators might also help to clarify the molecular mechanisms underlying several ciliopathies^55^. It is possible that Tβ4 regulates re-organization of actin cytoskeleton structures in biogenesis of primary cilia through ROS-Nrf2 axis. Therefore, it is required to examine how ciliogenesis could affect actin networks via downstream signaling pathways, vice versa.

Although many questions remain regarding the nexus between CC viability and Nrf2-mediated regulation of TB4 expression and PC formation, our findings suggest Nrf2 counter-regulates PC formation and TB4 expression to maintain CC viability (Fig. 6F). We cannot rule out the possibility that ROS-induced molecules might affect PC formation and TB4 expression. These results help clarify CC viability under SD conditions.

## Supplementary Information

Supplementary Information 1.

Supplementary Information 2.

## Data Availability

All data generated or analysed during this study are included in this published article [and its supplementary information files] and are also available from the corresponding author on reasonable request.
