# Supplementary material for: Nuclear factor (erythroid-derived 2)-like 2 counter-regulates thymosin beta-4 expression and primary cilium formation for HeLa cervical cancer cell survival
Source: Sci Rep. 2022 Nov 23;12:20170. doi: 10.1038/s41598-022-24596-6 (PMC9691707; doi:10.1038/s41598-022-24596-6)
Supplement: Supplementary file 1 — Supplementary Information 1. [file 41598_2022_24596_MOESM1_ESM.docx]

**Nuclear factor (erythroid-derived 2)-like 2 counter-regulates thymosin beta-4 expression and primary cilium formation for HeLa cervical cancer cell survival**

Jae-Wook Lee^a^, Pham Xuan Thuy^a^ Ja Hyun Koo^b^ & Eun-Yi Moon^a,^ *

^a^Department of Bioscience and Biotechnology, Sejong University, Seoul 05006, Republic of Korea

^b^College of Pharmacy and Research Institute of Pharmaceutical Sciences, Seoul National University, Seoul 08826, Republic of Korea

* Corresponding author: eunyimoon@sejong.ac.kr


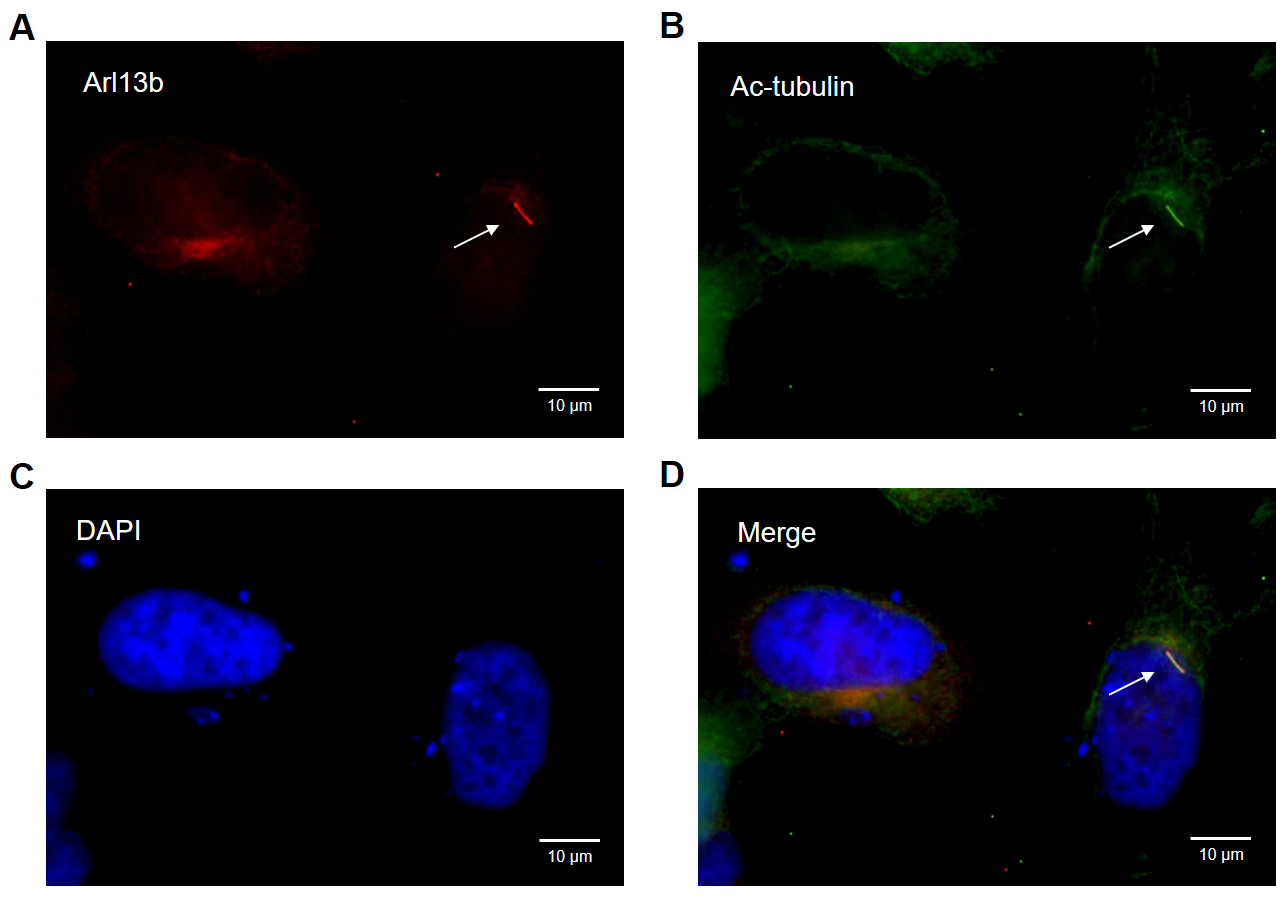


**Supplementary Figure S1.** Cilium was detected by immunostaining. HeLa cells were grown on coverslip and incubated with serum-deprived DMEM for 36 h. Cells were fixed with 4% paraformaldehyde for 10 min, washed three times with cold PBS, and permeabilized with 0.1% (v/v) Triton X-100 for 10 min. After washing three times, cells were incubated with anti-Arl13b antibodies (A) and anti-acetylated (Ac) tubulin antibodies (B) for 2 h at room temperature. After washing three times, cells were incubated with goat anti-rabbit IgG-Alexa 568 (A) and/or chicken anti-mouse IgG-Alexa 488 (B) for 1h at room temperature. Nucleus was visualized by staining cells with DAPI (C). After washing with PBS, cells were mounted on glass slide. Primary cilia were observed and photographed at 1,000 x magnification under a fluorescence microscope. Arl13b and Ac-tubulin respectively detected by Alexa 568 (red) and Alexa 488 (green) were merged in cilium (D). White arrows indicated primary cilia.
