# Supplementary material for: Nuclear factor (erythroid-derived 2)-like 2 counter-regulates thymosin beta-4 expression and primary cilium formation for HeLa cervical cancer cell survival
Source: Sci Rep. 2022 Nov 23;12:20170. doi: 10.1038/s41598-022-24596-6 (PMC9691707; doi:10.1038/s41598-022-24596-6)

Nuclear factor (erythroid-derived 2)-like 2 counter-regulates thymosin beta-4 expression and primary cilium formation for HeLa cervical cancer cell survival

Jae-Wook Lee[a], Pham Xuan Thuy[a], Ja Hyun Koo[b] and Eun-Yi Moon[a, *]

[a]Department of Bioscience and Biotechnology, Sejong University, Seoul 05006, Republic of Korea
[b]College of Pharmacy and Research Institute of Pharmaceutical Sciences, Seoul National University, Seoul 08826, Republic of Korea

Running title: Nrf2 counter-regulates TB4 and PC formation for CC survival

*Corresponding author
Eun-Yi Moon, Department of Bioscience and Biotechnology, Sejong University, 209 Neungdong-ro Kunja-Dong Kwangjin-Gu, Seoul 05006, Republic of Korea.
Tel: +82 2 3408 3768; Fax: +82 2 466 8768.
E-mail address: eunyimoon@sejong.ac.kr (E.Y. Moon)

Supplementary information

1. Corresponding author authorized that we checked western blots used in figures 4A, 4C, 5A, 5D and 6A for their compliance with the digital image and integrity policies (www.nature.com/srep/policies/index.html#digital-image).

2. Corresponding author authorized that we did not make the grouping of blots cropped from different parts of the same gel, or from different gels, fields, or exposures (e.g., using clear delineation either with dividing lines or white space).

3. Corresponding author authorized that we also did not make high-contrast (overexposure) of western blots. Blots are all from original blots by applying the process to change brightness and contrast equally across the entire image. For blots in figures 4A, 4C, 5A, 5D and 6A, processing (such as changing brightness and contrast) is applied equally to controls across the entire image. We have mentioned this in figure legend of figures 4A, 4C, 5A, 5D and 6A.

Figure 1B

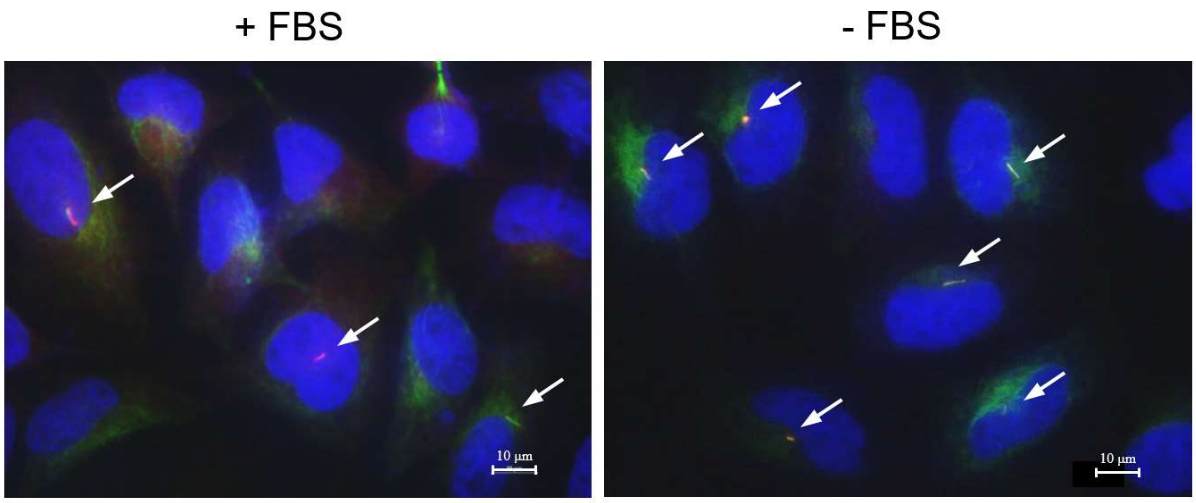

Figure 2C

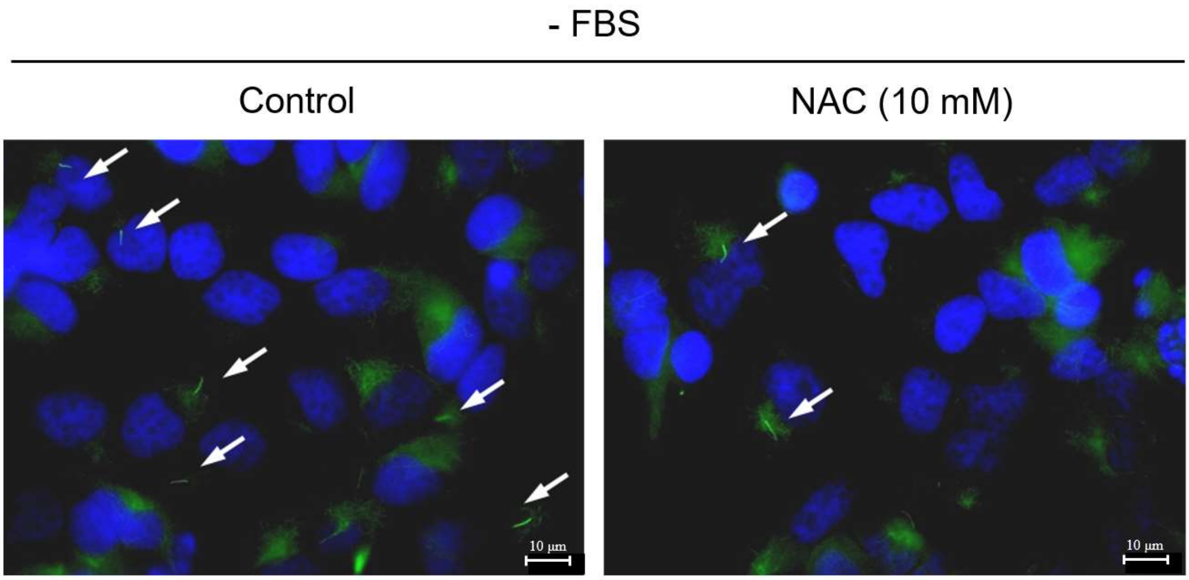

Figure 3A

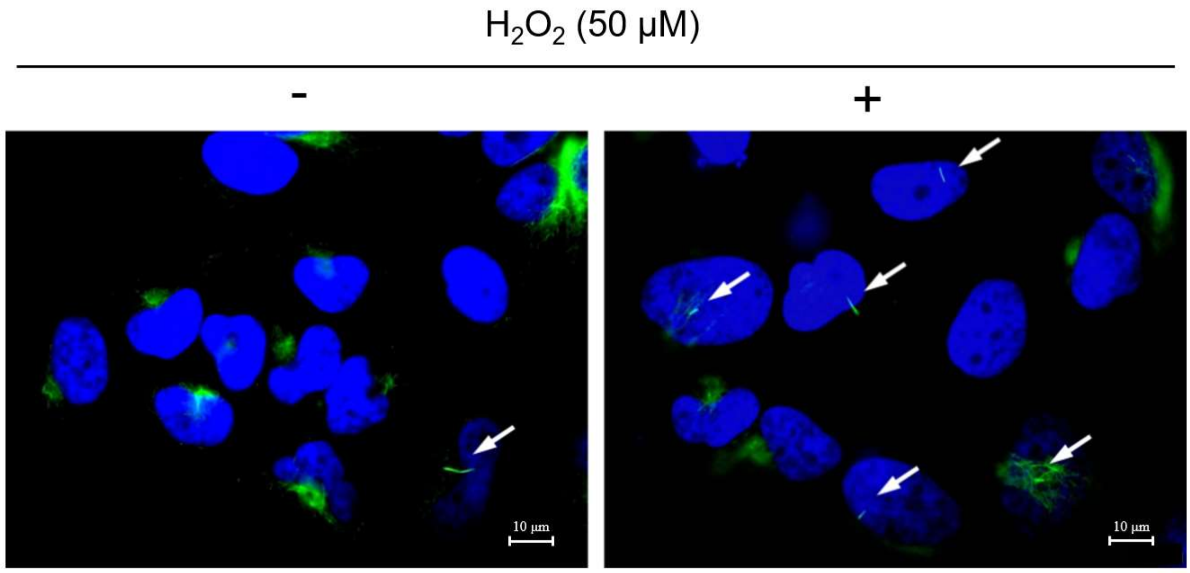

Figure 3B

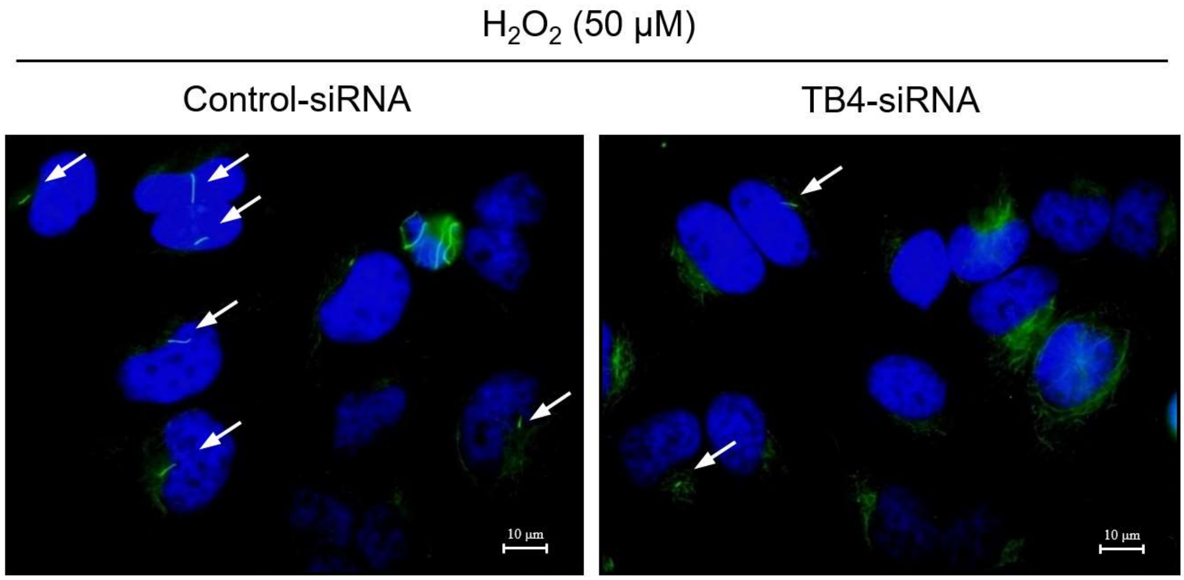

Figure 4A Origina

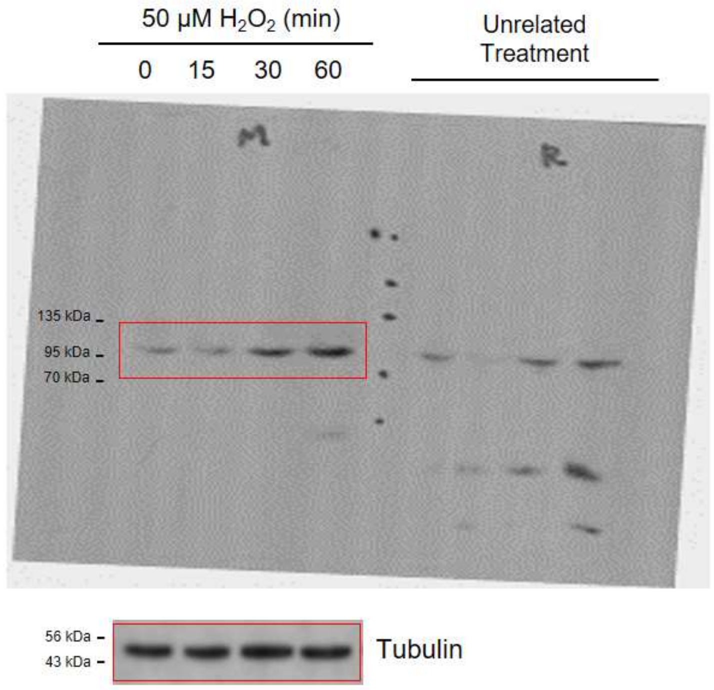

Figure 4C Original

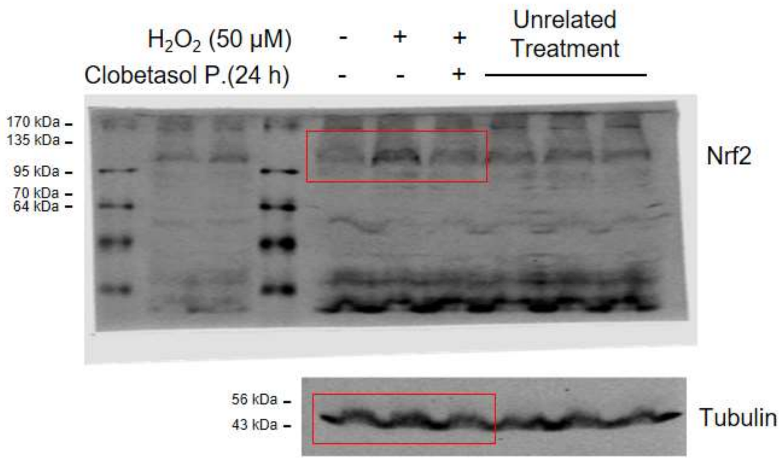

Figure 5A Original

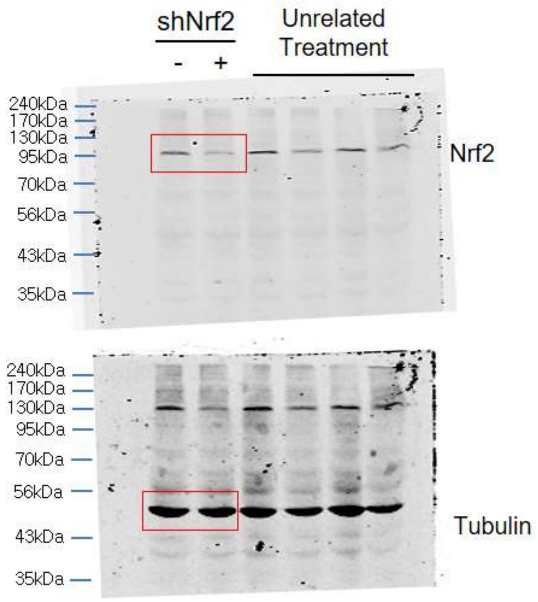

Figure 5D Original

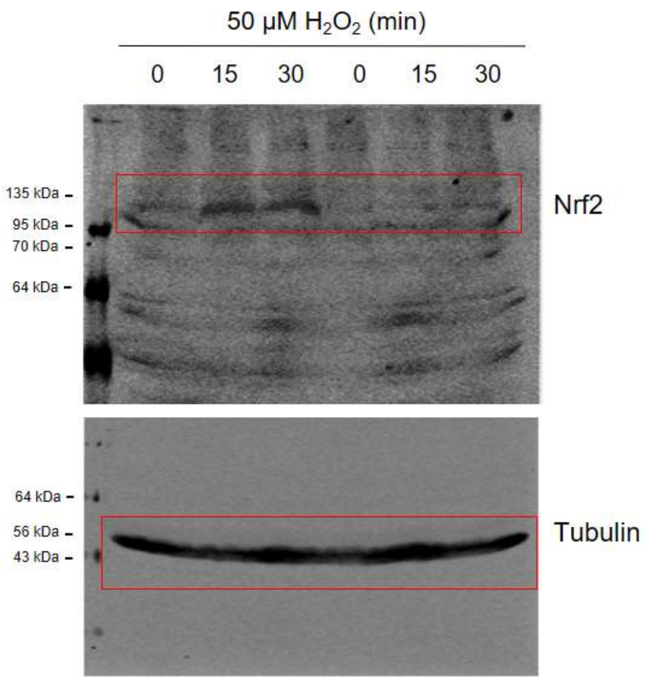

Figure 6A Original

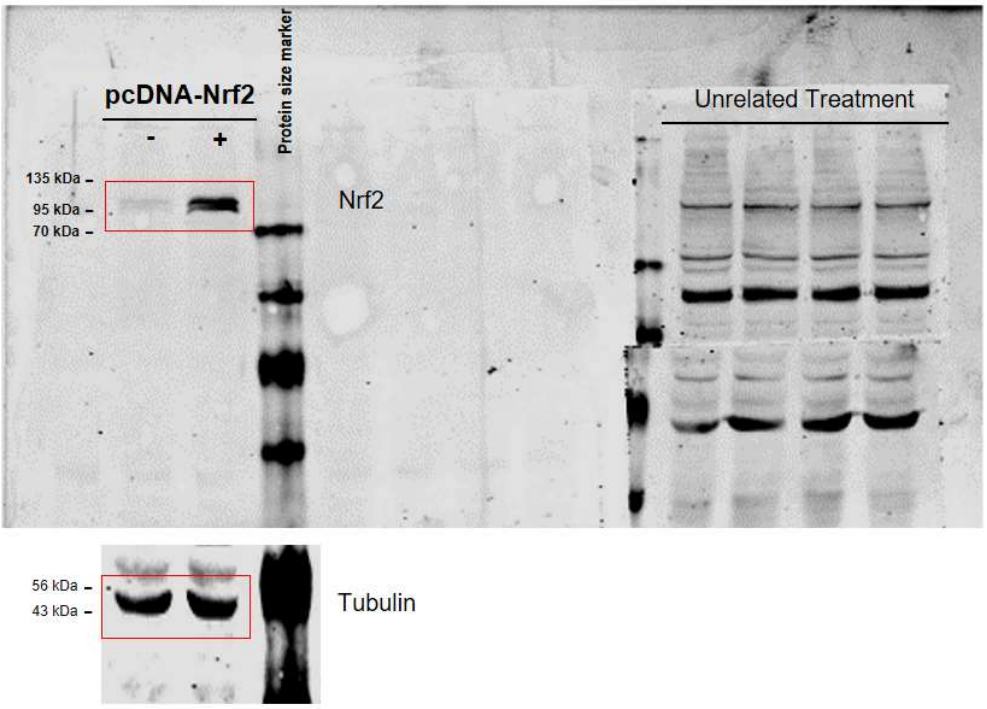

Figure 6D

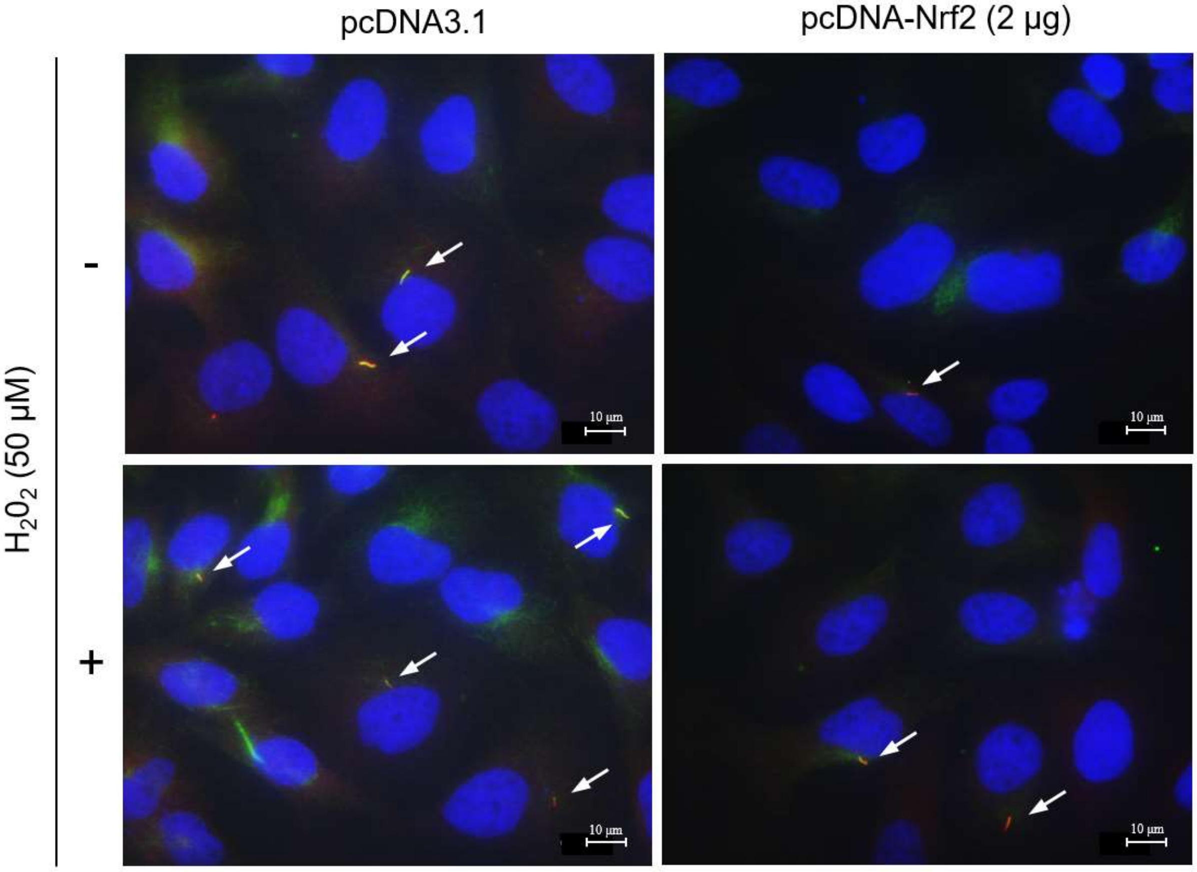

Supplement: Supplementary file 2 — Supplementary Information 2. [file 41598_2022_24596_MOESM2_ESM.pdf]
